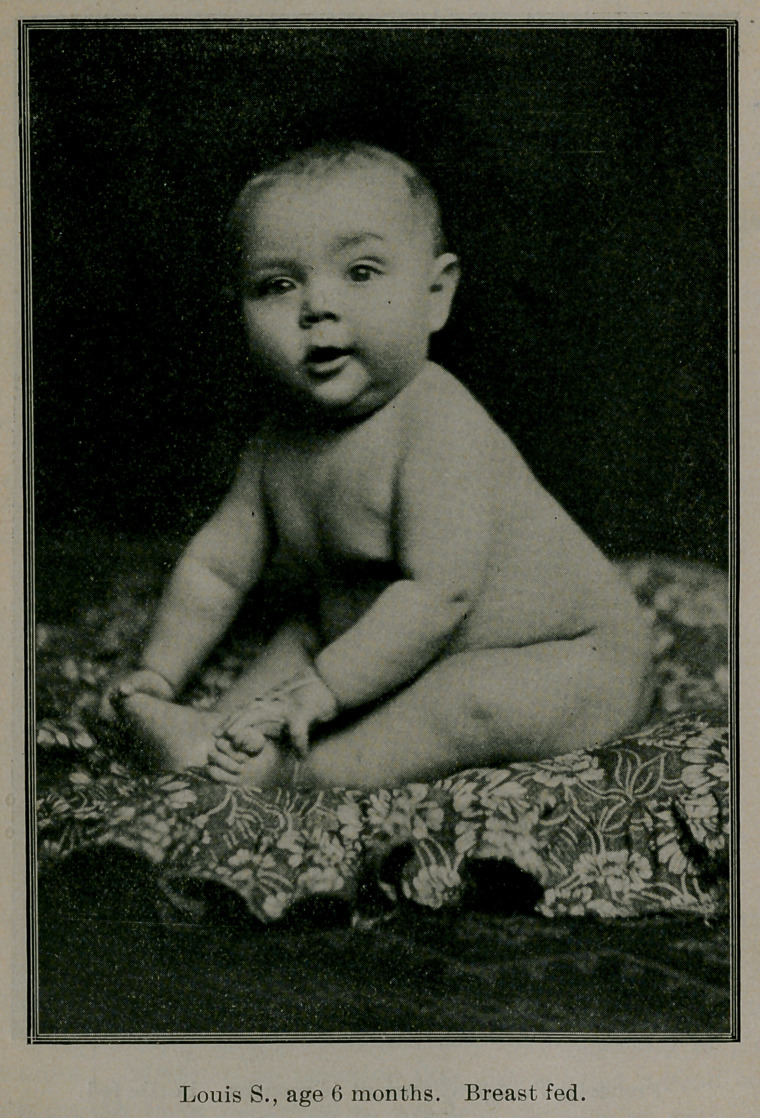# Infant-Feeding

**Published:** 1901-05

**Authors:** Samuel A. Visanska

**Affiliations:** Atlanta, Ga.


### INFANT–FEEDING.*

BY SAMUEL A. VISANSKA, PH.G., M.D.,

ATLANTA, GA.

This subject is, to my mind, the most important known to medical science, and the art of infant-feeding requires the greatest skill. It is most important, because right here we lay the foundation of future generations. Without properly fed infants we cannot grow strong men and women; "it is the rock upon which many and many a little bark has made shipwreck." How few of us seem to realize these facts when we inquire as to how infants are fed, and how the food is prepared! Experience has taught us that there are but two ways of feeding an infant, namely, either on human milk, at the breast of the mother or of a wet nurse, or upon an artificially prepared milk diet; and we have the two corresponding classes of the breast-fed and the bottle-fed baby. God in his infinite wisdom gave to the mother two glandular organs—mammæ—which secrete sterile milk, with all the properties necessary for the nutrition and growth of the infant. Happy are the little ones who belong to this class, for there is no question whatever, that the natural and proper food for infants is human milk. Statistics show, beyond a doubt, that breast-fed babies, as a class, are larger and healthier than the bottle-fed ones, and that the mortality of them is far less. The most careful preparation cannot possibly make the

*Read before the Medical Association of Georgia, at Augusta, April, 1901, milk of another animal chemically identical with that of a woman, or similar in its effects on the child. Therefore, it is the duty of every healthy mother to nurse her infant, and it is still more the duty of the attending physician to see that this method is carried out. As to the time a baby should be put to the breast, physicians differ. I make it a rule that as soon as the mother has rested sufficiently, and she and the baby have been washed, to put the little one to the breast. The natural instinct of the baby is to suck, and though there is little nourishment in the breast the first two or three days, still there is a thin, yellowish sticky fluid looking like poorly developed milk, called colostrum. This is of a peculiar character, since, besides being of a nourishing nature, it has a somewhat purgative action upon the child's bowels, and it is advisable that these be well opened early, and the blackish contents (meconium) discharged. It usually requires three or four days for the secretion of milk to become well established. (This is an important point to remember, as you will often find the primipara alarmed at not having an abundance of milk the first day.) The early sucking of the child fulfils still other purposes besides those mentioned. It both stimulates the secretion of milk and draws out the nipples into better shape for the baby's future use; it also insures proper uterine contractions. Many monthly nurses wish to feed the child during the first day or two of life with sweetened water, gruel or other substance. This is usually totally unnecessary, and often harmful. During the first six weeks of life the child should be put to the breast regularly every two or two and a half hours during the day, and from this, up to the time of weaning, every three or four hours. During the night it should be fed not more than twice. Too much stress cannot be laid on the importance of regularity in nursing. If the mother nurses the child every time it cries great harm results, as the baby's digestion is sure to suffer, and its disposition to cry to become more and more. Moreover, a baby is, to a wonderful degree, a "slave to bad habits"; it will make every one connected with it a slave to itself. It is a great mistake to suppose that every cry that stops temporarily during nursing denotes hunger; often the child is thirsty and a litte water is all that is needed, and it should be given often.

Turning, then, to another very important point is the necessity of early weaning. If the mother is suffering from the drain of nursing, as from general debility, consumption or other exhausting ailment, or if she should develop any severe acute disease, such as typhoid fever, pneumonia or the like, nursing the child is out of the question. It is very commonly believed that the return of the menstrual period makes nursing improper. Although you will find this occurrence very often does render the milk unfit for the child, yet this is not an invariable rule, and the mother should do nothing precipitately in the matter. After the occurrence of one monthly period there may be no reappearance of it for several months, and weaning would have been entirely unnecessary. Each case is a rule to itself, and only the effect upon the mother and baby can settle the question, even if the periods have returned regularly. Should the mother become pregnant again, the child should be weaned, for it is too great a strain upon her to sustain both, the one present and the one to come. Sometimes you find a great falling off in the quantity and quality of the milk, which cannot be remedied in any way, or there may be an entire or early cessation of the flow; again, the breast milk may be richer than the child can digest. Before breast feeding is abandoned on account of any defect in the amount or quality of the milk, the mother and physician should make sure that the trouble really exists, and that it cannot be remedied. The only accurate method of determining the character of the milk is, of course, to have a chemical analysis made of it. Observation, however, as to the growth of the child, and if it is gaining in weight will usually suffice. Crying is not always a symptom of insufficient food. If the child tugs long and hungrily at the breast, and is unwilling to cease sucking after it should have finished, or if, perhaps after a period of nursing it drops the nipple with a dissatisfied cry, it is very likely that the milk is insufficient in quality. Sometimes you will notice the milk is pale and bluish, even though very abundant, showing that the richness is interfered with. I came across a case the other day where there was an almost entire cessation of the flow of milk. This was due to a very anxious mother taking little nourishment, and being up night and day with her sick child. I did not suggest weaning, but immediately put the mother on a diet of milk and cream, with the addition of Hoff's Malt and Iron. In a few days the milk returned to its normal quantity and quality; the child, in the meantime, was also placed on artificial diet. As we often find some defect in the quantity and quality of the breast milk, the question arises whether something cannot be done to correct this instead of at once subjecting the baby to the trials of artificial feeding. Modification of the amount or kind of food taken, and of the method of living and the frequency of nursing exerts a powerful influence upon the composition of the milk.

Rotch gives the following table for modifying breast milk:

| | |
|---|---|
| To increase the total quantity: | Increase the liquids in the diet. |
| To decrease the total quantity: | Decrease the liquids in the diet. |
| To increase the total solids: | Shorten nursing intervals.<br>Decrease exercise.<br>Decrease the liquids in the diet. |
| To decrease the total solids: | Lengthen nursing intervals.<br>Increase exercise.<br>Increase the liquids in the diet. |
| To increase the fat: | Increase the meat in diet. |
| To decrease the fat: | Decrease the meat in diet. |
| To increase albuminoids: | Decrease the exercise. |
| To decrease the albuminoids: | Increase the exercise to the limit of fatigue. |

Should the mother find it necessary to abandon nursing, she does not necessarily have to wean the baby, since she may employ a wet nurse. There is no question that the milk of a good wet nurse is greatly to be preferred to artificial feeding, as you only exchange the milk of one mother for that of the other. Still, there are many objections to the employment of a wet nurse, and it is often impossible to obtain a suitable one. Nevertheless in the case of an extremely delicate child, the employment of a wet nurse often is the baby's only chance of life. I will not dwell on the methods of selecting a wet nurse, as they are, no doubt, familiar to

Frederick S., age at present 14 months. Bottle fed.

every physician. The principal point to remember is to see that they are free from specific or tubercular taint.

*Artificial Feeding.*—This brings us to the consideration of the very complicated subject of artificial feeding, one of the most perplexing problems which can present itself to the combined study of mother, nurse and physician. While science has done much for

Louis S., age 6 months. Breast fed.

surgery and other branches of medicine, the artificial feeding of babies is still in the background, no two physicians agreeing on this most important subject, and for this very disagreement many children are now lying under the sod. It is amusing at times, when getting a history of a case where a child is fed on artificial food, to hear the mother say, "How doctors do differ! Have had five doctors with this child, and each one orders a different food. Dr. A says preparation No. 1 will not agree with my child; Dr. B says 'take it off Dr. A's food or it will starve to death,' and so on." Is it a wonder the layman has begun to look upon us with suspicion? It is well, however, that we do differ at times as to diagnosis and treatment, but we often "agree to disagree" when there is no need for it. To feed a child artificially requires more skill than to perform the operation for appendicitis, yet you will see journals and text-books filled with articles as to the best method of performing this operation. How few articles we see on infant-feeding, and even then little attention is paid to them. Society has done much for the bottle-fed baby, from the fact that so few mothers of to-day will nurse their infants, preferring the ballroom and club, thereby placing a greater number of infants on artificial food, and naturally a greater number of physicians have to direct the preparation of same. In endeavoring to feed a baby properly, we must bear in mind three important factors:

1. The quantity of the food.
2. The quality of the food.
3. The individual peculiarities of the child.

(1) One of the most frequent mistakes made in feeding a baby is that of giving it a much greater quantity of food than it can possibly assimilate, with the result that it either vomits it or passes it through the bowels in an undigested state. A new-born baby's stomach holds without distention only about one ounce (two tablespoonfuls). How foolish then to direct to feed the new-born child from a full-sized nursing-bottle and allow it to gorge itself with all it will take.

(2). The quality of the food. Regarding the character of food to be given a child, that is, its quality, it is evident that the more closely the food resembles mother's milk, the more likely it is to agree with the child. Chemistry has almost solved the problem of artificial feeding by giving a thorough analysis of woman's milk and cow's milk, which should be familiar to every physician.

Rotch's table gives:

| WOMAN'S MILK. | COW'S MILK. |
|---|---|
| Reaction, alkaline. | Reaction, acid. |
| Bacteria, none. | Bacteria, present. |
| Water, 87.88 per cent. | Water, 86.87 per cent. |
| Total solids, 12.13 per cent. | Total solids, 13.14 per cent. |
| Fat, 4 per cent. | Fat, 4 per cent. |
| Albuminoids, 1 per cent. | Albuminoids, 4 per cent. |
| Milk sugar, 7 per cent. | Milk sugar, 4.5 per cent. |
| Ash, 0.2 per cent. | Ash, 0.7 per cent. |

Besides these characteristics, woman's milk has a specific gravity of about 1031, while that of cow's milk is about 1029. My experience has taught me that modified cow's milk is the *ideal* artificial food for feeding infants, and a point that I want to make clear is this, it is not so much how you prepare the food as it is what you prepare. For fear that this broad assertion might be misconstrued I will explain further. While I prefer and use sterilized milk for infants, the point is this: If you have the milk, cream, water, sugar, etc., in proper proportions, whether you sterilize, pasteurize or give the food raw, nine times out of ten it will agree with the child. Sterilization only destroys the bacteria and keeps the milk sweet for a longer time; it is only a process of steam heating. "And here's the rub." By what method are we to determine the proper ingredients for artificial feeding? We must have a starting point, and then modify to suit each individual taste, as you will find every infant "a law unto itself." Jacobi says, "An infant's stomach should not be made a test-tube." I say it is a great experimental station, provided the experiment does not last too long. The method I have adopted of feeding babies is that of Professor Siebert, and that is to feed according to the weight and not the age of the child. There is good reason for this; a large baby requires more food than a small one, just as a large horse requires more food than a small horse, and it is on this principle we should feed infants. To illustrate, a baby at the age of three months might weigh eighteen pounds, while the average is twelve and one-fourth pounds. If you were to direct the preparation for the three months old baby according to its age, the formula would be for a baby weighing twelve and one-fourth pounds; therefore five and three-fourths pounds of the baby would be insufficiently nourished. On the other hand, a child at the age of three months, weighing eighteen pounds, can be placed on the same formula as the average baby of eight months weighing eighteen pounds.

*How to Prepare the Food.*—Having adopted Siebert's method of feeding by weight and not the age of the child, we will take, as an example, the child weighing twelve pounds. Referring to Siebert's chart, we find: size of bottle, five ounces; milk two and a half ounces, water or gruel two and a half ounces, sugar one and one-half drachms; one bottle every two and a half hours. In twenty-four hours, seven botttles; 6 A.M. to 6 P.M., five bottles; 6 P.M. to 6 A.M., two bottles.

*Directions.*—Make use only of fresh bottled milk brought to the house every morning (including Sunday) early, by some reliable milkman. Never use milk from large cans, but have it delivered in bottles. Take the upper half of a quart bottle of milk (or when necessary of two) so as to include all of the cream, and pour into a clean pitcher, dissolve with sugar of milk, add a pinch of salt, if you like, and then filter through a small layer of clean absorbent cotton placed in a funnel, directly into the nursing bottles up to the milk line.

(2) *Filtration of Milk.* This filtration of milk and sugar water through a small layer of absorbent cotton, as devised by Dr. A. Siebert (Archives of Pediatrics, July, 1894), removes all gross impurities from these fluids and seven-eighths of the bacteria from the milk. The amount of cream in the milk is not diminished by this filtration; filth, manure, cow's hair, dust scales and bacteria (germs of disease) are present in all milk, even when delivered fresh and in bottles. The aluminum milk filter devised by Siebert is very convenient, and sold at 50 cents each, the cotton filter discs 25 cents per box.

(3) *How to Sterilize.*— Arnold's sterilizer is the best on the market, and comes in different sizes. See that the bottles are thoroughly cleansed, and after having filtered your milk solution into the nursing bottles, place a piece of clean raw cotton in the nursing bottle; pour hot water into the kettle up to the ridge, then place lid on top and steam over a brisk fire, according to the season, from May 1 to November 1, 30 minutes, and from November 1 to May 1, 20 minutes. Directly after feeding, rinse the bottles with clear warm water, and then fill them to the top with soap water, made with green caustic soap (or soft soap). The bottles remain filled with soap water until evening, when they are cleaned best by soap water and absorbent cotton wound around a stiff wire. Never use brushes. Remains of milk inside the bottles are best seen by holding them in front of a strong light and must be carefully removed. Nipples should be placed in weak soda solution and rinsed in clear water several times. If you find the child is constipated add a little cream to each bottle, just before placing the nipple on, and heat to 90°. Never let the baby sleep with the nipple in its mouth. It is not only a bad habit, but has a tendency to produce mouth-breathing. A very convenient chart for directing the feeding of an infant is the one devised by myself:

CHART FOR FEEDING AN INFANT.
Devised by
SAMUEL A. VISANSKA, PH.G., M.D.,
Atlanta, Georgia.

Name ..................................................
Weight ..................................................
Age ..................................................
Milk ..................................................
Water ..................................................
Cream ..................................................
Sugar of Milk ..................................................
Sugar White ..................................................
Salt ..................................................
Number of bottles ..................................................
Size of bottle ..................................................
One bottle every .................................................. hours
.................... bottles 6 A.M. to 6 P.M.
.................... bottles 6 P.M. to 6 A.M.
Nurse ..................................................
Date ..................................................

It is of no advantage to have the milk from one cow; it is in fact a distinct disadvantage, for the great difference which exists between the milk of different cows makes it impossible to prepare a proper imitation of mother's milk according to any fixed rules unless you should have the individual cow's milk analyzed in order to determine in just what way the mixture should be made. Besides this, the milk of any cow is subject to variations from time to time depending upon the nature of the food given it, the health of the animal and other factors. The selling of milk should be under the direct supervision of the respective Boards of Health, and each bottle should bear a label: "Certified milk," with official signature. I am glad to see a move in this direction in the different cities, and it would not be a bad idea to extend to the smaller towns. Owing to the rigid inspection of milk in New York City people dwelling in tenement houses can secure as pure milk as those residing on fashionable Fifth Avenue; what a godsend to the poor babies!

*Thickening Substances.*—Sometimes we find it necessary to add thickening substances, such as barley water, gelatine, etc. They act purely mechanically, by getting, as it were, between the particles of caseine during coagulation, preventing their running together and forming large compact masses. Barley water is made by adding a tablepoonful of "pearl barley" to a quart of water, boiling for fifteen minutes, strain and add water sufficient to make the quart. If an alkali is necessary to add to the milk, use lime in the shape of lime-water, or, preferably, the saccharated solution of lime; five to fifteen drops of the latter can be added to each bottle.

*Preparations on the Market.*—As to the preparations on the market, much could be written. Having stated fully my views as to the very best method of feeding infants, I say use them if you like, but you are not imitating nature. There is not a text-book that will advise them. Unfortunately they often agree with the infant, and sometimes they grow fat, but not strong, and if you watch the child's future, you will find they do not have the power of resistance necessary to overcome disease. We all agree that condensed milk will make fat babies, but I compare the fat of a condensed milk baby to that of the beer-drinker—it is soft, flabby, unnatural. Unless you understand how to feed infants, you will be unable to treat such cases as marasmus, rickets, scurvy, diarrheas, etc. Beginning with marasmus, Louis Starr says: "The chief etiological factor is diet. It occurs both in breast-fed babies and those brought up by hand, being in either case due to insufficient nourishment. The child wastes because he is starved. As I have already stated, food can be insufficient in two ways: First, when it is supplied in amounts too limited to meet the demands of the system. Second, when it contains a minimum of the elements essential to nutrition or presents them in a form ill adapted to the feeble digestive powers of infancy.

*Infantile Scurvy.*—William Perry Northrop, of New York, says: "The most frequent direct cause is prolonged feeding upon patent prepared foods and canned condensed milk, or, to state this point more directly, persistent deprivation of fresh food." He further states, "there is no evidence that sterilized milk has caused scurvy, nor any reason to believe that pasteurizing milk predisposes thereto."

*Rachitis.*—J. Lewis Smith of New York, says: "Besides the anti-hygienic conditions which give rise to rachitis, the most common and potent appear to be the use of food not sufficiently nutritious, or, if nutritious, not suited to the age and digestive powers of the child. The use of thin and poor breast milk and artificial food of poor quality or not suitable for the stage of growth and development, is a common cause of rachitis." He says further, "I might relate cases of rachitis occurring during the use of certain proprietary or commercial foods. I have examined the analysis of these foods made by Professor Leeds in order to determine what ingredient is lacking, and they are found to contain a considerably smaller percentage of fat than occurs in human milk. Too little fat in the food may, as Cheedle observes, be one of the chief dietetic causes of rachitis. It is an interesting fact, and one that throws light on the dietetic cause of rachitis, that it does not occur in Japan. Physicians who have had abundant opportunity to observe the diseases of the Japanese state that they have never seen or heard of a case among them, and Remy says that the Japanese women have a remarkable abundance of milk and that they suckle their young until the age of five or six years, but their children are also given other foods after the first year. Remy's explanation of the immunity of the Japanese from rachitis is as follows: The Japanese have always eaten plentifully of fat and oils of fishes, the blubber of the whale, the eel and loach, especially.

### REPORTS OF CASES.

To demonstrate my method of feeding infants, I will report three cases:

CASE 1. Louis S., age three months, weight 18 pounds, a perfect specimen of babyhood; his mother was suffering with a severe facial neuralgia and acute gastritis, unable to nurse the child. Louis was given modified sterilized cow's milk, according to Siebert's chart (feeding by weight), and the milk was entirely digested. As I stated before, the formula was the same as the average child of eight months. As soon as the mother recovered, the child was again put to the breast.

CASE 2. Frederick S., age ten months, was brought to me from Selma, Ala. At birth only weighed four pounds, always had a weak digestion. Had gone through an attack of pneumonia and whooping-cough. When I first saw the child it was extremely emaciated, head of a peculiar shape, typical of "marasmus"; skin harsh to the touch, and of a light brown hue; urinated every few minutes, and stools of a green color with foul odor and very frequent. On inquiry from the mother I learned that the child was fed on condensed milk and barley water, and it required two bottles full at each nursing to satisfy the baby. Is it a wonder the child urinated so frequently? The child weighed only eleven pounds, and I gave it the modified sterilized cow's milk with barley water, according to Siebert's chart, it being equal to the formula of the child age three months. To each bottle of milk I added 10 gtts. ess. pepsin, 10 gtts. liq. taka diastase, aq. menth. pep. 1 drachm. The milk was perfectly digested, and in 48 hours the stools became normal in color, odor and frequency, amount of urine also diminished. In a few weeks the child began to take on new life; the complexion cleared up, he gained in weight each week, and at present weighs 24 pounds. The flesh is firm, not flabby; has cut several teeth with no disorder of dentition. "Fritz," as we call him, is now a fine specimen of a baby boy.

CASE 3. Jenet W., age five months. History: At birth, weighed about eight and a half pounds. At the age of six weeks mother noticed child did not digest the breast milk; passed mucus, would fret all day, skin looked sallow. The child was treated from time to time, sometimes better, sometimes worse. Took her to Macon (home of mother), thinking perhaps the change would benefit the child, but it did not. Physicians there advised the mother to take baby to Gainesville, which she did, but the child was not benefited. The child had taken breast milk, malted milk, Mellins' Food, condensed milk, etc. About September 15 Mrs. W. returned to the city, bringing the child with her. On September 18, was called to see the girl. Found her very emaciated, almost a skeleton, bowels loose, of a greenish color, loaded with mucus, odor very offensive, very fretful, skin harsh and sallow. She weighed only eleven pounds; after taking her off of all manner of food except the barley water and "toddy," cleansing the gastro-intestinal tract for two or three days, I put her on the modified sterilized milk and barley water, according to Siebert's chart. To each bottle of milk I added 10 drops ess. pepsin, 10 drops liq. taka diastase, aq. menth. pep. 1 drachm. The milk was thoroughly digested, stools became normal in color and frequency, the child slept like all healthy children, fretting was a thing of the past, at first gained three-quarters of a pound a week and sometimes one pound a week. As she gained in weight the strength of the milk was increased and a little fresh cream added to each bottle. The girl is now in perfect condition, has cut six or eight teeth without any disorder, and at the age of one year weighs 21 pounds.

66½ *Whitehall Street.*

## Figures and Tables

**Figure f1:**
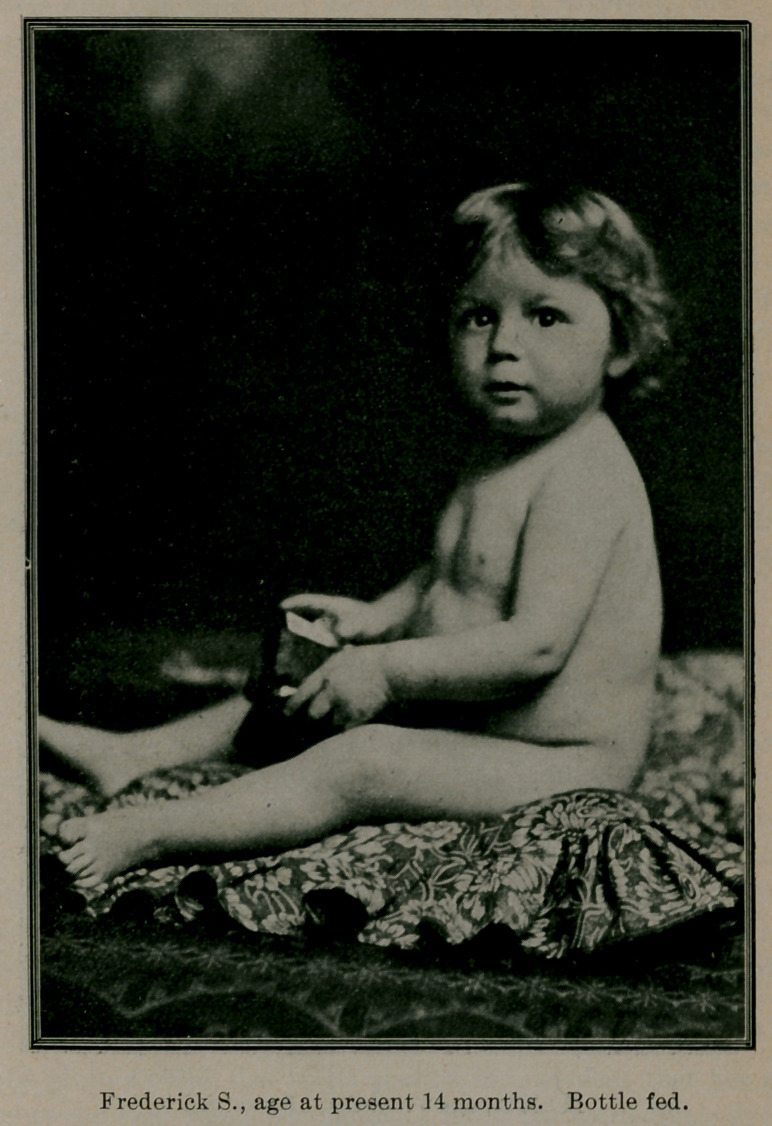


**Figure f2:**